# Sharing space at the research table: exploring public and patient involvement in a methodology priority setting partnership

**DOI:** 10.1186/s40900-023-00438-1

**Published:** 2023-05-02

**Authors:** Nikita N. Burke, Derek Stewart, Theresa Tierney, Andrew Worrall, Maureen Smith, Jim Elliott, Claire Beecher, Declan Devane, Linda Biesty

**Affiliations:** 1 Evidence Synthesis Ireland and Cochrane Ireland, University of Galway, Galway, Ireland; 2 School of Nursing and Midwifery, Aras Moyola, University of Galway, Galway, Ireland; 3 Honorary Professor, University of Galway, Galway, Ireland; 4 Public Co-Author, Health Research Board Primary Care Clinical Trials Network Ireland, Galway, Ireland; 5 Public Co-Author, Evidence Synthesis Ireland and Staffordshire, Staffordshire, UK; 6 Public Co-Author, Cochrane Consumer Network Executive, Ottawa, Canada; 7 Public Co-Author, Evidence Synthesis Ireland, Galway, Ireland; 8 Health Research Board Trials Methodology Research Network, University of Galway, Galway, Ireland

**Keywords:** Public and patient involvement, Patient engagement, Methodology, Evidence synthesis, Rapid review, Co-production, Priority-setting partnership, Mutual learning

## Abstract

**Background:**

Public and patient involvement aims to improve research quality, relevance, and appropriateness. Despite an increasing evidence base on the influence of public involvement in health research, the role of involvement in methodology research (i.e. research that aims to enhance the quality and rigour of research) is less clear. Using a qualitative case study, we explored public involvement in a research priority-setting partnership in rapid review methodology (Priority III) to give practical insights to inform public involvement in priority-setting for future methodological research.

**Methods:**

Participant observation, documentary analysis, interviews and focus groups were used to explore the processes of Priority III and identify the views and experiences of the participants of a steering group (*n* = 26) regarding public involvement in Priority III. We used a case study research design and conducted two focus groups with five public partners; one focus group with four researchers; and seven one-to-one interviews with researchers and public partners. Nine episodes of participant observation of meetings were conducted. All data were analysed using template analysis.

**Results:**

The findings of this case study present three themes and six subthemes:

Theme 1: We all bring unique qualities to the table.

- Subtheme 1.1—Coming from different perspectives towards shared-decision making;
- Subtheme 1.2—Public partners bring pragmatism and grounding in reality;

Theme 2: We need support and space at the table.

- Subtheme 2.1—Define and develop support needed for meaningful involvement;
- Subtheme 2.2—Creating safe space to listen, challenge and learn;

Theme 3: We all benefit from working together.

- Subtheme 3.1—Reciprocity in mutual learning and capacity building;
- Subtheme 3.2—Relationships as partners in research, with a feeling of togetherness.

Communication and trust, as inclusive ways of working, underpinned the partnership approach to involvement.

**Conclusions:**

This case study contributes to knowledge on public involvement in research by explaining the supportive strategies, spaces, attitudes and behaviours that enabled a productive working partnership to develop between a team of researchers and public partners in this research context.

**Supplementary Information:**

The online version contains supplementary material available at 10.1186/s40900-023-00438-1.

## Abstract (summary)

Public and patient involvement is well known in research where patients share their lived experience for a health-related study. However, the role of public and patients in methodology research (research that aims to improve the quality of research) is not clear.

A priority-setting partnership brings patients, carers, clinicians and other stakeholders together to jointly identify priorities for research. We looked at public involvement in a priority-setting partnership in how we plan, do, and share the results of rapid reviews—the Priority III project. We wanted to do this to better support public involvement in future research.

We explored the processes of Priority III and asked the members of the Priority III steering group for their views and experiences of public involvement in the project. We found three themes:

1: *We all bring unique qualities to the table.*

2: *We need support and space at the table.*

3: *We all benefit from working together.*

Communication and trust were found to be important across all themes. Even though public partners felt outside of their comfort zones when starting the project, they significantly helped the project, brought unique views, ideas and practical solutions. Support and safe spaces were needed to help overcome challenges due to the complex methodological concepts. Researchers and public partners learned from one another, and developed relationships with a feeling of being “partners in research”. Our findings offer insight into what helped public involvement in this research context. We give examples of practical actions and suggestions for future research.

**Supplementary Information:**

The online version contains supplementary material available at 10.1186/s40900-023-00438-1.

## Introduction

Public and patient involvement (PPI) in research aims to improve the quality, relevance, and appropriateness of health research [1]. PPI can and should occur across the research cycle—from prioritisation to implementation [2]. However, approaches to public involvement can be varied, and uncertainty still exists in the research community on how “best” to “do” involvement. Involvement in research can at times be at risk of being tokenistic—for authentic and meaningful involvement, there must be a clear purpose with the aim of improving the project and ultimately, enhancing health and wellbeing [3]. Real-world perspectives can increase the relevance and value of research; involvement can improve research processes, transparency and impact; and the public have a right to be involved, as research is in the public interest [4].

There are increasing reports in the literature describing various approaches to public involvement in health research [5]. Such reports encourage reflective learning and help to share methods that work in certain contexts.

This paper presents a case study of public involvement in the Priority III project [6]. Priority III was a Priority Setting Partnership (PSP) conducted to identify the top ten unanswered questions for future research on how we plan, do and share the results of rapid reviews in healthcare. A rapid review is a type of evidence synthesis that brings together and summarises information from lots of different research studies to produce evidence for people such as the public, researchers, policy makers and funders in a systematic, resource-efficient manner [6]. Priority III was conducted by Evidence Synthesis Ireland (www.evidencesynthesisireland.ie), based at the University of Galway, in collaboration with the James Lind Alliance (JLA), a non-profit organisation that brings multiple stakeholders together to identify the most important evidence uncertainties about specific topics.

In this case study, we aimed to explore the views and experiences of the researchers and public partners of Priority III and document the processes of public involvement used. Instead of recommending a particular involvement method, this approach involves sharing learning and practical insights. We did this to support future research teams who want to involve the public in priority-setting for methodological research. We hope this study adds to the body of evidence on how research can involve key stakeholders in a useful, meaningful and authentic way.

The evaluation of the impact of public involvement in research [7] has been criticised because the tools and methods used give precedence to performance indicators that matter to researchers and not to the public [8]. This is often examined as a one-way exchange of information and does not account for the mutual learning of researchers and public partners. We draw on Knowles’ [9] call for "*comparative case studies which explore differences in research approaches and different research contexts*”, Tierney et al [5], who encourage reporting of accounts of involvement to promote innovation and appraisal, as well as Staley and Barron’s’ [10] conceptualisation of public involvement as “*conversations*” between the public and researchers that support two-way learning.

### Context

The Priority III Priority-Setting Partnership (PSP) [6] project involved the convening of an international steering group, an initial survey, an interim survey, a consensus workshop, and a dissemination phase. Members of the steering group (*n* = 26) comprised public partners (5), researchers (6), clinicians (6), policymakers (4), funders (3) and representatives of the JLA (2). As PRioRiTy I [11] and PRioRiTy II [12] were focused on methodological uncertainties rather than on treatment uncertainties, a modified JLA approach was developed, to address methodological uncertainties within randomised trials. By contrast, Priority III related to methodological uncertainties in rapid reviews, a concept considered technically-complex to public partners, compared to a health-related topic. Public partners were paid for their time on the Priority III project, in line with INVOLVE guidelines (2013) [13].

Of the five public partners that were members of the Priority III steering group—three were previous members of either the PRioRiTy I or PRioRiTy II steering groups, one was recruited through Cochrane and another through a local PPI group, using existing connections or networks. The five patient and public partners possessed varying knowledge and experience of rapid reviews and methodological PSPs.

### Aim

This qualitative case study aimed to explore public involvement in the Priority III Priority-Setting Partnership, to give practical insights to inform practice in public involvement in priority-setting for future methodological research.

## Methods

### Rationale for this case study

This qualitative case study was carried out at the request of, and with, the public partners of Priority III, as they felt that there was a benefit in reflecting on and sharing the learning from Priority III. All participants thought something about this experience was different to other studies they participated in and wanted to explore that further. They thought that this knowledge would benefit other research teams, that it would have been useful to have explored and reported public involvement in PRioRiTy II, another methodology PSP, which might have subsequently informed Priority III. The public partners framed it beyond an “evaluation”, in that it was more important to capture *how* researchers and public partners worked together (processes) and reflect on what both groups learned.

All public partners contributed to the prioritisation, conception, design, conduct, analysis and writing of this case study, and were also paid for their time on this study, in line with INVOLVE guidelines (2013) [13]. Due to the travel restrictions in place during the COVID-19 pandemic, both the Priority III project and this case study were conducted online using Zoom videoconferencing software.

### Definitions

We use the term “*public partners*” to describe the public, patients, and caregivers who are involved in shaping research in partnership with researchers, and *public involvement* to describe research carried out with or by public partners rather than to, for or about them, and research that is carried out ultimately for the public good.

“*Research team*” refers to the Principle Investigator and the Project Lead who led, coordinated and guided the activities of Priority III project. *Methodologists* refer to the researchers with expertise in rapid reviews who participated in the Steering Group of Priority III.

### Study design

A single intrinsic case study research design was chosen to support an exploration of the views and experiences of the researchers and public partners of public involvement in Priority III. Case study research underpins the exploration of a unique (*intrinsic*) phenomenon (*the case*) in its real live context [14]. Described by Yin as an “*intense focus*” on events, case studies can help to *explain, describe or explore* a contemporary phenomenon, particularly when the phenomenon, and the context in which it occurs, are intertwined [15].

The *case,* in this study, was defined as public involvement within the Priority III project (specifically in the Steering Group). Given the unique phenomenon of Priority III in that it was a PSP focusing on methodology, this single intrinsic case study is distinguished [16] or different from other PSPs that may focus on a health-related topic (e.g. asthma, hypertension). However, the learning reported in this paper may be useful for future research teams.

### Participants

Priority III Steering Group members were invited to participate in this case study, and all members took part. The five public partners took part in two focus groups. A purposeful sample of four methodologists participated in another focus group. Three researchers, one member of the JLA and three public partners participated in one-to-one interviews.

### Ethical considerations

Ethical approval was obtained from the Research Ethics Committee at the University of Galway (Reference Number 20-Apr-02). Participants consented to participation in both Priority III (see Beecher et al [6]) and this case study. Participants were informed about and consented to the observation aspects of this case study. Participants were informed that though the Priority III Steering Group membership would be public, all data would be anonymised and not linked to individuals.

### Data collection

Three sources inform this study of evidence—observation, documentary analysis, and interviews (focus group and one-to-one interviews). Data for this case study were collected between September 2020 and March 2022 by NB and LB.

#### Participant observation

The first author of this case study (NB) was also a member of the Steering Group of the PSP project and conducted participant observations during Priority III “pre-meetings” and full Steering Group meetings. Pre-meetings were online meetings held prior to the main Steering Group with the research team of Priority III and the public partner subgroup (*n* = 5 individuals, total meetings *n* = 5, see Additional file 1: Data collection timepoints). Observations focused on public partner’s roles, language and communication, managing conflicting perspectives, decision-making, valuing contributions, influence, etc. Observations were recorded in the form of field notes and facilitated by an audio recording of each meeting. Eleven hours of participant observations were conducted by NB.

#### Documentary Analysis

All minutes associated with meetings and email and process documents were included in the documentary analysis (about roles, language and communication, decision-making, contributions, influence, etc.). This data source helped to describe the processes employed related to public partners and how these are reflected in the minutes and documents. Fifty-eight documents were included for analysis (See Additional file 1).

#### Interviews

All public partners were invited to participate in a one-to-one or focus group interview(s) at specific time points during Priority III (Additional file 1). A purposeful sample [17] of other members of the Steering Group were invited to participate in either a one-to-one or focus group interview to establish their experiences and views of public involvement in the PSP (Additional file 1).

The interviews were semi-structured with an open-ended approach to facilitate sharing of unanticipated ideas and experiences [18]. The thematic structure of the interview guide was informed by previous studies exploring stakeholder participation in health research [19, 20]. The topics explored included roles, expectations, support, and relationships, with space to raise additional issues (See Additional file 2: Interview and Focus Group Topic Guide). Focus groups lasted 60—67 min. One-to-one interviews lasted between 28 and 77 min. All interviews were facilitated by audio recording and transcribed verbatim. NB facilitated one-to-one interviews, and focus groups were facilitated by NB with support from LB.

### Data analysis

Data analysis was an iterative process in that we looked across the data sources to identify the broad issues that the case study participants noted as important to their experiences and used template analysis, a seven-step form of thematic analysis, to organise the issues into themes to present our findings in a useful manner [18, 21].

The steps of template analysis were first applied across the interviews and focus groups. For the first step, *familiarisation with the data,* two researchers (NB, LB) read and re-read transcripts. Both researchers then highlighted the relevant units of information (data) they believed provided an insight into the aim of this study and aligned a descriptive label (a code) to this data (*coding,* step two). For the third step, *clustering*, the researchers organised the codes into groups/clusters of broad themes using tables within Word documents, an initial version of the template, consisting of the broad themes, was generated (step four). For the fifth step, NB used the template to a guide analysis of a selection of the documentation and observational data, and LB peer-reviewed this. This process supported modifications to the template to ensure it reflected all the broad themes noted across the different data sources (for an example see Additional file 3). The template was then applied across the data set of this case study by NB, in that all data were coded to the broad themes, this step was peer reviewed by LB. Adding relevant field notes and raw interview data to the template document identified context to the findings and helped NB and LB to refine to three overall themes and generate subthemes which provide a further exploration of the concepts contributing to each theme. This version of the initial findings, was presented to the remaining co-authors for review and feedback. This wider review of the analysis and interrogation of the findings contributed to the rigour of the case study methods. The public partners wanted to meet to reflect on the findings and agree on how they would be presented. This meeting also enhanced the rigour of the case study and was instrumental in ensuring that the findings were agreed across all the co-authors as a collective representation of learning. This collective review also ensured that the findings did not preference the views of any of the different groups. The final step seven of template analysis, i.e., interpretation of the data, is presented in this paper.

### Reflexivity

The specific rationale for conducting this case study has been identified in this paper, and the co-authors have noted their desire to capture and share the learning during Priority III. All co-authors of this paper (bar LB) drew on their “insider” views and experiences of Priority III, which is evident in the presented findings. LB was not a member of the Priority III Steering Group. Her contributions to this case study are methodological and provide an “outsider” viewpoint to the data collection, analysis and presentation of the discussions noted in this paper (see “Context” section).

Given that some of the co-authors of this paper also contributed to the data, we acknowledge the importance of reflexivity throughout. The roles of all co-authors in this case study were agreed in advance. Participants were aware that participant observations would be used as a form of data collection and the impact of this on group dynamics was discussed (and deemed not to inhibit interactions). As noted previously, NB and LB undertook data collection and the initial steps of data analysis. In keeping with the traditions of reflexivity within qualitative research [22], NB and LB had frequent discussions exploring the rationale for the decisions they made and how they interacted with the data and particularly in relation to generating and describing the findings. Strategies undertaken by the co-authors of this case study (e.g. the co-authors meeting to interrogate further and discuss the presentation of findings) is an example of a planned reflexive moment embedded in this study to contribute to the integrity of our process.

## Results

**Fig. 1:**
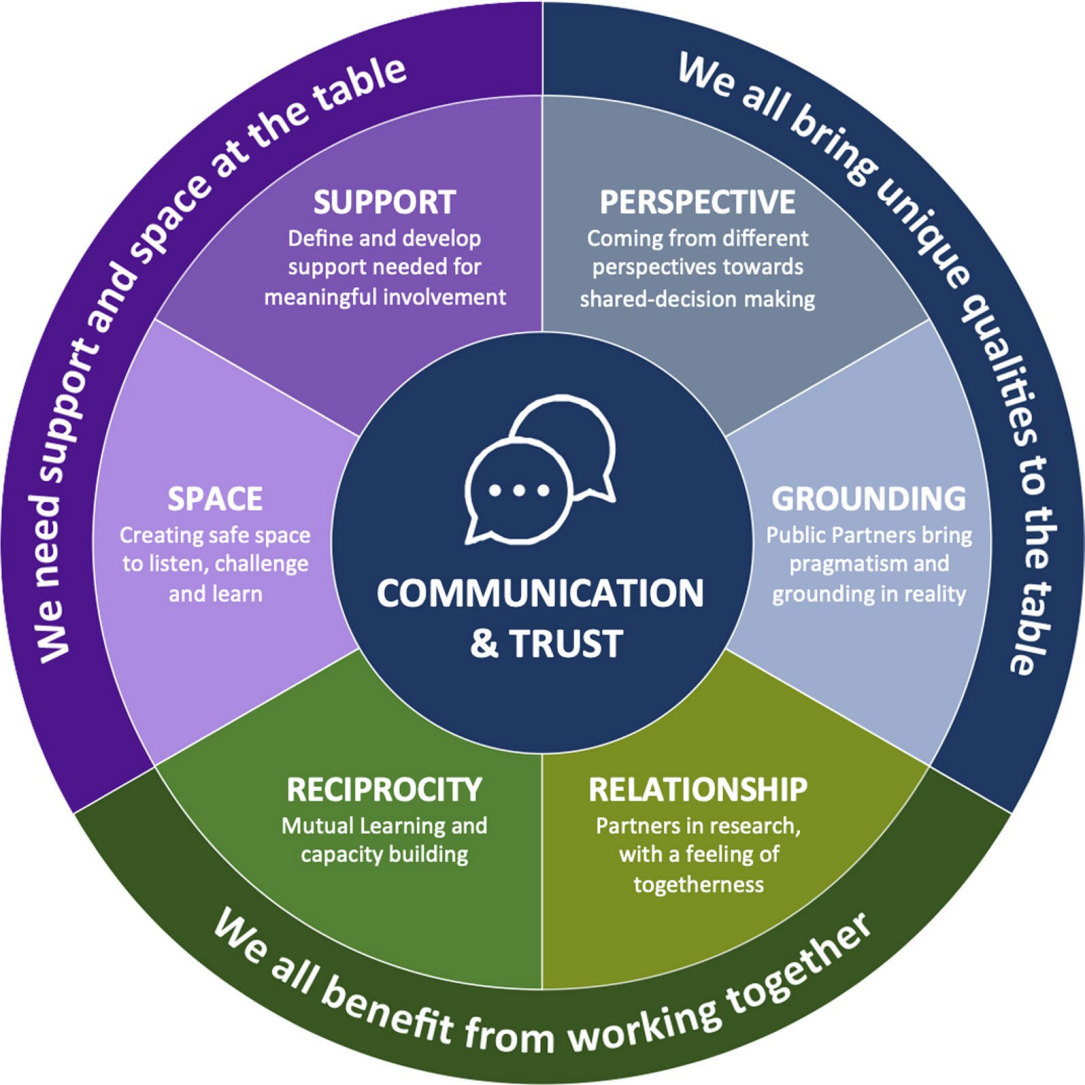
Themes and subthemes

The key findings of this case study present three themes and six subthemes (Fig. 1). The theme title and a broad summary describe each of the findings, with subthemes to explore different, but inter-related aspects of the theme. The findings are presented as a narrative description of the themes, with illustrative examples from the data, that capture the learning identified by participants of this case study. While we present these in a linear order that helps us to illustrate the experiences of the participants, all themes presented are of equal importance. In the theme headings, the word “we” refers to the public and researchers, working in partnership.

The label of two themes and indeed the title of this paper draw on the metaphor of a table. We are aware that some research teams may not physically meet and may never sit together in a room, at a table. However, we suggest that the table is a representation for the ways in which we gather and come together as a research team, and welcome people to work in a collaborative manner.

### Theme 1: we all bring unique qualities to the table

The research team was initially uncertain how best to involve the public in Priority III, a PSP in rapid review methodology. The research team engaged a group of public partners that purposely had differing levels of experiences concerning the context of Priority III: some had evidence synthesis experience, some had prior experience of PSPs, and some had experience in neither PSPs nor evidence synthesis. This theme highlights the importance of a range of different perspectives in shaping the PSP's operationalisation, through challenging assumptions, seeking clarity and bringing practical solutions.

#### PERSPECTIVE: coming from different perspectives towards shared-decision making

When they were first approached, public partners spoke of their uncertainty about how they could contribute. They stated that they felt outside their comfort zone given the topic. When reflecting after the completion of Priority III, some public partners recognised that their novice status brought a fresh perspective. By asking clarifying questions and seeking accessible language, public partners initiated discussions that challenged the assumptions held by the methodologists.

Interview, Public Partner 003 “*I feel what I can contribute is that question around whether a layperson should be involved in conversations that are unrelated to their specific experience. In terms of my own work, I look at things from a communications perspective. And sometimes, if I even just ask the right question, it might get the researcher to think in a different way. So even if I don’t have any idea but I just seek clarity, sometimes it creates a conversation that wouldn’t have happened otherwise.”*

Researchers recognised that they held assumptions that the public partners challenged. This influenced their thinking, how they articulated their discussions in meetings, and their communication with the broader participants of Priority III. Ultimately, public partners' challenges led to decisions that made the process clearer for all. Researchers stated that they valued the public partners perspectives and noted that, at times, the direction of Priority III changed due to the discussions (see Additional file 4: for a description of activities and where public partners influenced the project).

An example of constructive discussion occurred when public partners strongly advocated for a need for definitions of rapid reviews, evidence synthesis, and systematic reviews. Some of the methodologists felt that the existing, traditional definitions were sufficient. Public partners highlighted that these definitions were not accessible and then worked with researchers to reach a consensus over meetings, email, and working documents until a shared understanding was reached.

However, shared decision-making did not always mean agreement, and all participants of this case study referred to this. This was not a point of criticism: what was viewed as more important was that all opinions were considered, that decisions were transparent and were clearly, honestly and frequently communicated with the Steering Group.

Focus Group, Public Partner 004*. ‘When we said, well—what do you actually mean by rapid review? There was a deep breath and there was an hours lesson which, in the end of it I was thinking, what if we hadn’t asked that fundamental question? Now, we didn’t get a perfect answer, but actually we got involved in the discussion around why there isn’t a perfect answer*.’

Throughout this case study, public partners acknowledged differences and expressed their respect for the methodologists’ perspectives—they also commented that they thought this respect was mutual. This regard for the opinion of others was thought to contribute to the decision-making process: all voices were heard, all opinions were considered, and there was transparency in decision-making.

Focus Group, Public Partner 003: “*I’ve learned that everyone has some perspective to share and, even if I spent a lot of time at the start just listening and learning and growing my knowledge of the context, that it’s a really respectful atmosphere. No one should feel that they don’t have enough to say or enough background to not become part of the process.”*

#### GROUNDING: public partners bring pragmatism and grounding in reality

A key quality that public partners brought to this project was pragmatism—bringing solutions and grounding the project in reality. This was recalled in focus groups, interviews and noted during the participant observations. Public partners brought accessibility in relation to language and communication, and solutions that helped how Priority III evolved. These issues of pragmatism, grounding, feasibility and accessibility are separate yet interconnected matters. The pragmatic perspective of public partners often led to frank conversations, illustrated in the below examples.

At the start of Priority III, public partners were concerned with some of the language provided in the PSP information. They wanted to spend time teasing out the issues to ensure clarity. This altered timeframes and meant that original milestones were not met. Though these “delays” were perceived as unexpected by researchers, they acknowledged that addressing this issue enhanced the Priority III project through improved clarity.

In addition to the practical solutions that public partners brought to or prompted in the operationalisation of the project itself, researchers noted that public partners had an insightful understanding of the tension between what was “optimal” and what was “feasible” during Priority III in terms of progress of the project.

Interview, Researcher 002: *“…there’s almost a surprising contribution of the public partners to the Steering Group in terms of a focus on feasibility or pragmatism. I didn’t expect that. You’ve got public partners saying, well hang on a second, we need to take a step back from this, it would be ideal if we could do this, but [research team] have said that the impact of that would be four, five weeks. So let’s think about could we all live with this?”.*

Such tensions were sometimes noted, for example, when some researchers wanted the “right” solution to what constituted an answered question in the literature, contrasting with pressures of needing to reach project milestones with limited resources. Both groups acknowledged a tendency of researchers to bury down into, and get lost in, the detail, and that the public partners, with an eye to the bigger picture (in terms of the need for the project to be completed on time and under budget), helped move the process along.

Steering Group, Public Partner 001: “*Okay, so I’m one of the public partners and I’m struggling with this. It seems to me that since the kind of questions this whole process is about, is to do with trying to pull out those unresearched areas. Surely we don’t need to go any further than reviewing what systematic reviews already exist? Because you’d then be beginning to answer the kind of questions that we wish to put out there. And it just seems to me that there’s a bit of a danger of going in a never ending circle here.”*

In this way, public partners understood the time limitations and the project's need to add value. They suggested and supported practical ideas to ensure progress.

### Theme 2: we need support and space at the table

The research team strived to support the public partners to facilitate meaningful and purposeful involvement in Priority III. There were various types of support that the research team provided (Table 1). These were reported to have provided space, which facilitated meaningful contribution, enabled public partners to learn from each other and grow their confidence, and built trust with the research team and with each other.

**Table 1:** Description of various types of support that the research team developed, with feedback from the public partners, to facilitate meaningful involvement in Priority III

Support provided	Description
Group of public partners	There were five public partners on the PSP. The public partners felt that having more than one or two public partners was particularly needed given the methodology context
Payment policy	The research team with public partners developed a clear and transparent payment process that highlighted tasks the public partners would contribute to and how much they would be paid (Additional file 5)
Pre-meetings	Separate group meetings were held with the five public partners, the research team, and a representative of the JLA. The Principal Investigator of Priority III chaired these “pre-meetings”. These meetings were one hour with a break between the pre-meeting and Steering Group meeting. Initially, these meetings provided information around the topic and allowed space for questions and discussion. The agendas for these meetings mirrored the main steering group agendas in addition to any topic the partners wished to discuss
Individual email support	The research team offered a point of contact to the public partners and invited ad-hoc one-on-one support and group conversations in email threads to facilitate shared learning
PPI Item on Steering Group agenda	Initially, the group decided together not to have a PPI item on the agenda, lest it feel like a tick-box exercise. The public partners later requested to return the item to the agenda to facilitate transparency at the Steering Group of their activities and to provide an opportunity for questions. Each public partner presented these updates in a rotating manner
Pairing methodologists and public partners to review questions	A methodologist and public partner volunteered to review and refine the interim survey questions and then sent their collated feedback to the research team. All five public partners volunteered and participated in this exercise

#### SUPPORT: define and develop support needed for meaningful involvement

All participants thought that processes needed to be established early in the project's lifetime to support purposeful engagement, given the technically-complex nature of the project topic. The research team attempted to facilitate this by establishing several types of support (Table 1). The public partners also brought peer support, governance, and sharing of resources, for example. Researchers sometimes struggled with balancing attempts to share knowledge rather than reinforcing the idea that as ‘the experts’ their knowledge was the ‘right’ knowledge.

Interview, Researcher 002: *“I’m afraid of it not being that level playing field. I’m afraid of it being disproportionate power differentials going on. I’m afraid that the public partner won’t have a voice with the strong methodologists. I’m afraid of inadequate chairing of the groups and not giving voice to them. I’m afraid that no matter how good the chair is, that it’s impossible to create that level playing field or make that meaningful, purposeful.”*

All of the participants of this case study spoke of the specific and contextual information support that was needed to facilitate involvement. Flexibility was needed in terms of what specific, contextual support was needed for each stage of the PSP and for each person, as all had varying levels of needs throughout. Though some public partners felt that more context and background could have been given at the start, the personalised support was viewed as helpful and necessary. It gave context to otherwise complex methodological concepts.

Interview, Public Partner 001: “*I was very much a novice, and when [researcher] approached me, I did ask for some guidance and help and they pointed me in the direction of a couple of examples of rapid reviews. That was very useful. I felt I understood—well not enough—but sufficient to get by.”*

The “pre-meetings” were discussed at length during interviews and focus groups. Initially, the pre-meetings helped the public partners understand terminology and concepts in evidence synthesis and oriented them to the context of Priority III itself. Later, they were viewed as dynamic—a space for sharing resources and knowledge, seeing the big picture in terms of appreciating the relative significance of rapid review methodology in the context of people’s lives and reflecting on learning.

Another process to facilitate involvement noted in interviews and focus groups was pairing the methodologists with public partners to work on a part of Priority III. Each pair reviewed a portion of the questions generated from the initial online survey. The pair were tasked with reviewing if the submissions were in scope, interpreted and grouped correctly, or if the meaning of each summary question was unambiguous, for example.

Public partners spoke of challenges and benefits from this process, such as being initially intimidated by the volume of data, feeling unsure how to contribute, experiencing challenges with perceived power dynamics, and moving towards learning, finding it a fun, rewarding and useful process.

Focus Group, Public Partner 004: ‘*I said [to myself] why did I agree to this, and I closed down the file, breathed, went back and then thought, let me concentrate for an hour and get my responses together. It was only then that I felt confident enough to speak to [methodologist]. Then suddenly it made sense. But even with my experience and knowledge, I’m going bloody hell, I’ve overstepped the mark.’*

Methodologist participants also found this pairing exercise a useful process, where each pair came from very different perspectives and needed to work together to reach a consensus. Like the public partners, they also found it fun, interesting, and a learning experience.

In addition to these processes, the public partners often spoke of peer support: listening to concerns, sharing problems, and helping each other to understand the project, for example. Many references were made to the pre-meetings as being vital to support this. Having five public partners with varying levels of experience meant that different skills supported personal development within the group. Reference was made to how the pre-meetings helped build confidence, created a sense of togetherness, and built trust within the group. It was felt that this extra peer support was particularly needed given the methodology context but may also have a place within other research activities.

Focus group, Public Partner 005: ‘*When you’re in something which is perhaps outside the comfort zone—you can’t bring a specific lived experience to methodology research. So it’s thinking more what do I bring? That can seed doubts and then you might not speak up. Whereas we’re encouraged by others, we’re all very frank. So I think it’s [peer support] possibly more important in areas that aren’t related to your lived experience than it is in areas that are.’*

In terms of support, the research team stated that in hindsight, they might spend more time planning at the start. The research team felt tensions around ensuring specific types of support had purpose and were not overburdensome to public partners. They believed that the extra time it took to establish clarity on the project was essential to enable meaningful involvement. Though intensive and unexpected, it had the additional benefit of familiarising the group with the terms and language of the project and helped establish a shared sense of purpose.

Interview, Researcher 002: *“[regarding pre-meetings] I think it’s made a big difference… I see increased confidence in the public partners in engaging and contributing to the Steering Group. I see that they seem to be more prepared to engage in conversations around some of the nuances of rapid reviews, with the methodologists in particular.”*

Developing these processes required anticipating challenges, and planning time and space to allow for communication while also needing flexibility and iteration throughout.

#### SPACE: creating safe space to listen, challenge and learn

The types of support listed in Table 1 and described above created space, not just for public partners, but for the research team. The concept of space, both tangible space “at the table” in meetings (to ask clarifying questions, to challenge methodologists), and the space created outside of meetings (email conversations, time and space allowed for changing direction), created an environment in which trust was established. There was metaphorical space in terms of openness, flexibility, room for all opinions, and willingness to change.

Focus group, Public Partner 001 “*I felt there was enormous receptiveness of public involvement in this. It certainly wasn’t a question of having to vociferously advocate. One of the lovely things about it was right across this international group there seemed to be a ready acceptance of a need to listen to a wide range of stakeholders.”*

Public partners spoke to the importance of the space to learn, listen and reflect, in ensuring they could contribute meaningfully. This allowed the public partners to learn from each other and grow confidence in contributing to the wider project.

The research team spoke of the challenges and benefits of the slower pace set by public partners. Though this was not the pace that researchers were used to (as milestones had to be met), the space allowed dialogues to develop, constructive disagreements to be aired, and transparent conversations to be held, all leading to shared decision-making and an improved project.

Interview, Researcher 002. *“The public partners have been official brakes onto the Priority III process overall. They have slowed us down to the benefit of the product at the end of the day, the quality within it and what will happen at the end. It’s undoubtedly been better.”*

It was thought this pace was required given the context and brought authentic involvement to Priority III. The research team spoke of the benefits of this pace, to reflect when feeling challenged in the project. The tensions served as a learning edge for resolving key issues and recognising how to build better partnerships in future. The space helped them to adjust to new ways of working with the public and welcome alternative perspectives.

### Theme 3: We all benefit from working together

Through valuing different perspectives and creating space to discuss, everybody (researchers and public partners) gains from co-producing research. There is mutual learning with unexpected benefits.

#### RECIPROCITY: mutual learning and capacity building

Reciprocity was evident between methodologists and public partners and within the group of five public partners. In terms of their learning, public partners said they ultimately learned the importance of methodology research to people’s lives. They gained a greater awareness of evidence synthesis. There were examples of impact beyond the project, where a public partner linked up with a methodologist on the Steering Group to develop training on rapid reviews to support public involvement in their own country. Some public partners spoke of having more confidence when contributing to other methodological projects. Given the right context, they could not only contribute but also learn.

Meeting: Public Partner 002:* “I learned a lot about the methodology about rapid reviews, it gives you confidence to get involved in other methodological studies like PRISMA or core outcomes sets. In the right environment you realise, I can really learn and I can make a substantial contribution.”*

The project benefited from strong recruitment of public and patient participants to the survey (for example, 17% response rate from the public) and increased diversity of voices at the final consensus meeting (e.g. recruiting participants from low-to-middle income countries).

Researchers gained new perspectives to improve communication, data collection and interpretation.

Interview, Researcher 002: “*Simple things like we now have a much clearer definition of what a rapid review is because of the public partners. We’re now able to communicate much more clearly because of the public partners. We re-think, they continuously push us in that direction*.”

Researchers benefited from conversations with the public partners, in terms of finding solutions to challenges, getting support with forward action of the project, for example. Researchers also spoke of their learning as something they gained personally from the project:

Interview, Researcher 002 “*I personally have learnt an awful lot on this journey. We tried to be open to that learning; that’s been one of the biggest steps we’ve had to take. And to bring everybody with us*.”

As the public partners group consisted of varying levels of experience, this allowed for mutual learning within the group:

Focus Group, Public Partner 003: *‘If I was a lone wolf on this group I don’t think I would have lasted. Even just having the understanding interpreted through your eyes has helped me. When something is too big for my beginner’s mind to even wrap my head around, when I hear the other [public] members ask something, it puts it in a different context for me than the researchers would. And it might click, ah that’s what they’re trying to say, that’s what everyone else seems to understand’.*

Capacity building was considered from the start to ensure inclusion of people who were less familiar with research concepts and who might bring different perspectives compared to more experienced public partners, and who could then be involved in future studies and contribute to wider methodology research. The team recruited new public partners and some they had worked with before.

Interview, Researcher 002: “*Because there’s a risk of becoming dependent on a smaller number of people who are known to you. But it also gives the opportunity to meet some new people that you’d work with again.”*

Overall, everyone gained from working together, with two-way exchange observed and highlighted by participants.

#### RELATIONSHIP: partners in research, with a feeling of togetherness

Public partners spoke of mutual respect, feeling valued, and the importance of developing trust, which led to a sense of being “partners in research” with a feeling of togetherness, which was observed and expressed by the public partners and methodologists.

For example, when discussing the payments, public partners said that being asked for their feedback on the proposed payment helped them feel valued. They also found it useful to see the tasks comprehensively broken down, time estimated and costed individually, and suggested that others would benefit from this example (Additional file 5).

Focus group. Public Partner 002*: ‘[in terms of the payment process] I would add the breakdown of the tasks but also the opportunity to comment on it. So here is what we propose and what do you think about it, is it fair. That was a first for me, usually there’s no opportunity to comment. So I thought that was very respectful. Breaking it down [by tasks]—you feel as if somebody is taking the trouble to think of what your needs are”.*

Some public partners stated that they felt in safe hands to get involved with this project based on positive expectations from previous experience with the research team, highlighting the importance of continued relationship building (Additional file 6).

Interview, Public Partner 001: *“I guess it's also down to having had previous contact with you all, I had a sense that this would be a comfortable setting in which to try something which is well out of my comfort zone, because let’s face it, I know very little about methodology in research and specifically very little about evidence synthesis.”*

The research team had extensive negotiation with the publishing platform for the PSP protocol to have the public partners recognised in the manner they wanted. The article submission form required mandatory completion of institution. The public partners felt strongly that there should be the choice of having solely “public co-author” that was not affiliated to an institution. They felt this would make clear that their roles was as a member of the public, and expand the idea that publishing is for academics only.

Pre-Meeting, Public Partner 002:* “It’s really important for me to be identified as a patient partner. I would never entertain putting down an institution because I’m not an academic and I don’t want people to identify me as an academic. When I look at an article I always look to see who is the PPI in it, and sometimes I can’t tell. And I think that’s really bad. I’m not doing this to be melted down into a sea of academics. I want to be identified for what I’m doing.”*

The research team advocated on behalf of the public partners to change this, which was granted by the journal. This example illustrated the public partners’ leadership and served to demonstrate the partnership approach in the team.

There was an observed and expressed sense of co-production, with leadership also observed from the public partners in building capacity in others, prompting reflection and the sharing of learning, recruiting participants, and moving forward the project. All participants of this case study described a sense of relational openness, such as researchers and public partners being open to listening, respecting each other’s views and changing perspectives, that led to mutual learning between public partners and researchers, with reciprocity evident.

Public involvement approaches, as well as processes such as priority-setting activities, aim to balance power dynamics between researchers and public partners. However, it was also acknowledged that it can never be a level playing field, as everyone has different levels of experience. Even though the processes were laid out by the research team, their openness to change led to a mutually beneficial experience whereby public partners shaped the project's direction, and influenced the methodological quality, relevance, appropriateness and accessibility of the PSP.

Focus Group, Public Partner 004 *“I must speak to the leadership of [research team] who are open and willing to change. I remember [researcher] going ‘oh I need to think about that’ and then came back with a very open and transparent discussion about how we get to the next point. And I think that’s still quite unique and that’s been vital, it makes us feel a sense of belonging and trust that we’re part of something”.*

This case study highlights the learning gained in relation to public involvement in PSP of rapid review methodology from the specific context of Priority III. Communication and trust, as inclusive ways of working, underpin the partnership approach to involvement: communication between research team and the public and within the public group; trust in each other, and in the process. From the outset of Priority III, a research agenda was set, an endpoint concerning the study’s outputs was promised, and the research team was responsible for meeting the research goals. However, rather than researchers driving the project's trajectory, the experiences shared during this case study speak to the iterative learning and processes that were put in place to guide everyone to share space at the research table.

Focus Group, Public Partner 003. “*Listening to all of the different PPI people and the researchers was a huge learning experience for me. It definitely served to grow my confidence as well. Including not be afraid of looking foolish when asking something. And the kindness and respect that everyone has shown me throughout. It helped grow my trust in the system and the people involved for future groups.“*

## Discussion

This case study explores the shared learning of both the researchers and public partners of Priority III that could inform future methodological PSPs. Valuing a range of different perspectives and providing support and space facilitated and benefitted the project, the researchers and the public.

### We all bring unique qualities to the table

Our findings show that public partners have a legitimate and valuable role in a methodology research project, where lived experience may seem less relevant, and the role of the public may initially seem less intuitive. Most emphasised by participants was the unexpected role of being an outsider, both the fresh-eyed reviewer and the critical friend [23]. In these functions, public partners brought a pragmatism in holding the project to account. These perspectives should be valued, respected and explicitly communicated when recruiting partners—the public are there *because* of their distinct perspectives and different type of knowledge [24]. Their freedom to ask seemingly naïve questions [23] challenges assumptions that researchers may make and ensures greater clarity of methodology research.

These findings contest assumptions that public involvement in methodological research is ‘too technical’ or ‘too different from experiential forms of knowledge’ for a non-technical audience to understand or meaningfully contribute to. However, it is important to ensure that those around the table have a shared understanding of the concepts and definitions used, which would be expected to change within different contexts. Table 2 provides some considerations which research teams could use to support this. Our results show that public partners can not only contribute but also learn, and grow in confidence to contribute to future studies.

**Table 2:** Considerations when planning involvement for a methodology priority-setting partnership

*When recruiting public partners, consider*
A diverse mix of perspectives from novice to experienced in the topic area
Capacity building to include new voices, those who can think beyond their own immediate experience, and some who are experienced and can translate what researchers say
Communicating that prior experience in methodology is not a pre-requisite, and that the fresh perspective is welcomed
Explicitly acknowledging that the role of the public partner is to ask questions, challenge, see the big picture and bring pragmatic solutions
*Ways to support meaningful involvement might include:*
A comprehensive payment policy, including tasks individually timed and costed, and shared for feedback with public partners
Holding pre-meetings before Steering Group meetings to allow for information sharing, learning and relationship-building
Pairing a methodologist with a public partner to review data (and providing guidance around this)
Peer support, to include a diverse mix of experience in public involvement
*Researchers should:*
Be open and willing to step outside their own comfort zones
Plan early, ensure adequate time and resources, and be flexible
Communicate with public partners clearly, honestly, and frequently
Create space to allow for two-way exchange and ongoing dialogue
Invest in relationship building
*Public partners should:*
Recognise that their novice status is a distinct benefit to the project
Be prepared to ask clarifying questions and challenge researchers
Seek accessibility in relation to language and communication
Suggest pragmatic solutions to support inclusivity and forward action
Recognise the importance of peer support for mutual learning within public partners

To support inclusion in a research area where public partners may not bring a lived experience, we highlight the importance of having a group of public partners with a range of experience to support novice members. Having people who can think beyond their own immediate experience, interpret and translate what researchers say, and take on a more strategic role may be particularly important in research where lived experience becomes less significant [23]. Our findings in rapid review methodology may be relevant to other types of non-clinical research.

### We need support and space at the table

In Priority III, specific types of support were needed “at the table” to facilitate meaningful involvement. This support was an attempt to distribute power, and although it is not possible to create a completely level playing field, it helped facilitate conversations and create shared understanding. The physical and metaphorical space created an environment that built trust with the research team.

This concept of space frequently appears in the literature on public involvement [9, 24–26]. Bryant et al [27] studied a mental health research partnership and reported the importance of space in terms of tangible time to create a shared vision and space in attitudes and openness. Knowles et al [9] conceptualised space as “space to talk” and “space to change”, regarding the need to create space for dialogue by recognising the distinct contributions of the public and researchers and the need for two-way exchange. Space to talk, and space to change, were both evident in our study. Knowles [9] found that relational openness was perceived to be more critical than any particular approach to involving the public in itself, which should be considered when planning involvement.

Liminal spaces are described as those between or outside the normal roles of patient or researcher [24]. As argued by Maguire & Britten [24], for involvement to be perceived as authentic, researchers need to move into a partnership role and take a reciprocal step outside their own comfort zone. Stepping into the space created in Priority III invited researchers and the public to be present in a reflective place where all were uncertain, and needed to find creative ways to move onward together. This was a powerful way to engage in communicative action [24] that enabled a shared sense of purpose and forward motion.

The findings of our study add to the conceptualisation of involvement as operating within spaces which can be conceived as venues of facilitating communication [24]. Focusing on effective, respectful, two-way communication can support meaningful involvement in methodology research. Though we suggest that prior planning may be useful, it should be emphasised that responsivity and flexibility are needed to adapt, with continued communication, and to iteratively innovate as needs appear throughout the process. The creation of space (and allocating resources) to allow for this is a necessity.

The pre-meetings were emphasised as a critical support mechanism which created space. The purpose of the pre-meetings of Priority III aligns with the Dialogue Model of involvement [28], which acknowledges power differences and assumes that the public needs a separate, safe environment to develop their voice which then equips them for dialogue with researchers. This contrasts with the “traditional’’ James Lind Alliance approach for PSPs, where all groups, by design, have equal voice. Separate spaces may be particularly needed in the context of methodological PSPs. Indeed, failing to acknowledge differences in power, capacity or knowledge can be a barrier to authentic involvement [29], as despite the best intentions of equality, somebody in the group (most often the academic) has greater ownership of the space [28]. Therefore, providing spaces to overcome these barriers may facilitate authentic involvement in various contexts.

Sharing power was challenging as the research team had overall responsibility of the project, a reality of an academic context. These professional norms, the control of the agenda and responsibilities that brings can create an unlevel playing field [30–32]. It can therefore be difficult to share ownership of decisions—and as highlighted in this study, there was an understanding that decisions needed to be made, but also a recognition of the importance of communication throughout. The research team actively worked to address power imbalances to ensure effective involvement in this methodology PSP. We have highlighted ways to support such involvement in Table 2.

In terms of practical support, payment for time was emphasised as an important support in Priority III. The research team invited feedback from the public partners on the draft payment proposal. This early recognition of the contributions of public partners helped them feel like valued team members. In agreement with McMenamin et al [3], public partners should be invited to give feedback on payment policies, and our study provides a worked example to estimate tasks in a methodology PSP.

In Priority III, peer support of having five public partners with varying levels of experience was key—with the space to learn from each other and grow confidence in contributing to the wider project. This extra peer support was especially needed given the methodology context but may also be relevant to other research studies.

Together, safe spaces, preparation and support help the issue of ‘feeling out of my comfort zone’ for both researchers and public partners by providing space for people to express their discomfort, be reassured that they are not alone, to learn from one another, and come up with useful solutions to move forward together.

### We all benefit from working together

In Priority III, when perspectives were valued, and space was created, it led to meaningful involvement with reciprocity evident. There were benefits not just to the project but to the public partners and researchers, who all spoke of mutual learning. Participants' accounts align with co-production principles, such as sharing power, including and valuing all perspectives, collaboration, respect, open discussion, reciprocity, and relationship building [33, 34].

The research team emphasised the productive forces of conversation, tension and difference of opinion and perspective. Researchers valued how the project improved with public involvement. They spoke of their learning as something they gained personally from the project. Researcher’s learning is less typically reported in the literature, with those that do producing rich accounts of transformative experiences [3, 9, 10]. As well as benefits, there were some challenges, such as requiring extra time and careful explanation [24], which methodology researchers should be cognisant of. Indeed, co-production requires distinct skills, knowledge, and strategies compared with traditional research, and researchers must build capacity in novel ways of doing business [34].

Developing research partnerships needs to be underpinned by an ecosystem of mutual respect, creating spaces where public partners feel empowered to contribute [35, 36]. Methodology research environments may not initially feel like “safe” spaces given the technically-complex concept. Researchers need to invest in building relationships to facilitate authentic involvement [9]. Traditional ways of working in academia are often closed and rigid due to funding and ethical approval constraints [37], but is imperative to embrace uncertainty, create space and allow flexibility for iteration, especially in methodology research.

Part of creating safe spaces is building trust, which is essential when there are existing power differences [38]. In methodology research, which is less familiar to public partners, investing time in developing trust may be even more important. Building trust relies on power-sharing, fair distribution of resources, effective two-way communication, shared decision-making, and valuing different perspectives [38]. To create safe, collaborative spaces, it is imperative to create a respectful atmosphere, hold regard for the opinion of others, encourage questions and welcome being challenged. This will require, as described by Renedo and Marston [26] “ongoing spatial negotiation” between researchers and public partners, depending on the specific context.

In this case study, public partners told us that having their voices heard, and having transparent communication helped them to have a sense of belonging, and partnership. It should be noted that in Priority III, there were pre-existing relationships between the Principal Investigator and some of the public partners, which may have influenced trust building. However, in addition to having experienced partners, the team wanted to ensure fresh voices and to build capacity to mitigate the risk of becoming dependent on a small group of people who could become overburdened. Knowles et al [9] also worked with contributors with established relationships, acknowledging that this helped build trust, and argued that the academic norm of “refreshing” public partners is at the expense of long-term relationships. In this paper, we provide a model to do both—capitalise on existing relationships while inviting new voices to the table.

Our findings echo Staniszewkska et al [34], who highlighted that creating the optimal conditions for co-production requires “safe spaces where challenge can take place”, with enough time for open, honest, quality dialogue that considers power dynamics and manages tensions of pressures of funding, deadlines and research outputs successfully.

This paper gives one approach to involvement in methodology research. As emphasised by Staley and Barron [10], there is no one method of involvement but a variety of approaches that must be adapted to different situations and requirements of those involved.

### Methodological considerations

The Priority III project and the data collection for this study was conducted entirely online during the COVID-19 pandemic. Restrictions of online communication may have limited some aspects of conversation but also allowed contribution from a wider geographical location than would have been possible otherwise. The necessity of conducting Priority III online may have excluded some participants who were not digitally enabled, particularly those from underserved groups.

Some of the public partners involved were particularly experienced in public and patient involvement, which may influence the transferability of the findings of this study. As described, the research team balanced this by recruiting a group of public partners with a range of experience. As stated by Barker et al [23], public partners need not be representative of specific patient groups as the significance of the “outsider” role does not decrease even if the partners are at “professional” stage, as they are still untethered to academic limitations.

The co-authors of this case study drew on their own experience and insights to contribute to the findings. Using this approach is a strength of this study and provides an in-depth exploration, using multiple data sources, that may not have otherwise been possible. The co-authors as participants of and contributors to this study does not imply that it was not conducted rigorously. The principles of case study research and the qualitative methods used are detailed and the conduct of this study was supported by a qualitative researcher external to Priority III. While learning from this study is not generalisable, it may be transferable to other settings.

Equality, diversity and inclusion data was not collected for this case study and is therefore a limitation. Public partners were from Ireland, the UK and North America. Economic assessment, quantitative evidence of impact or robustness of measures were not assessed in this study [39] (see Additional file 6 for the completed GRIPP 2 checklist).

## Conclusion

When conducting methodological research, how, when and why to involve public partners may not seem immediately obvious to researchers or the public. Both parties may feel uncertain as to how to ensure meaningful contribution. This perceived barrier may hamper involvement and reduce the relevance of methodology research.

We have shared our learning and offered practical insights to inform practice in public involvement in priority-setting for future methodological research topics.

The case study contributes to knowledge on public involvement in research by highlighting the supportive strategies, spaces, attitudes and behaviours that enabled a productive working partnership to develop between a team of researchers and public partners. We hope the findings of the case study contribute to the existing literature on the effective and authentic engagement of the public in research.

## Supplementary Information

**Additional file 1:** Data collection timepoints. Table - List and dates of data collection time points in this case study.

**Additional file 2:** Interview and Focus Group Topic Guide. Semi-Structured one-to-one interview/Focus Group Schedule for Steering Group members of the Priority III PPI qualitative case study.

**Additional file 3:** Codes and subthemes. Table—Example of coding used and subthemes generated in this case study.

**Additional file 4:** Tasks and areas of influence of public partners in the Priority III PSP. Table - Description of tasks and activities that the public partners participated in and influenced throughout the stages of Priority III.

**Additional file 5:** Estimated task breakdown and time costings for the Priority III PSP. Table—Payment schedule and tasks.

**Additional file 6.** The GRIPP-2 Long Form is submitted as an appendix to this manuscript.

## Data Availability

The datasets used and/or analysed during the current case study are available from the corresponding author on reasonable request.

## Abbreviations

ESI: Evidence Synthesis Ireland
JLA: James Lind Alliance
PPI: Public and patient involvement in research
PRISMA: Preferred Reporting Items for Systematic reviews and Meta-analyses
PSP: Priority setting partnership
NIHR: National Institute for Health and Care Research in the UK
