# Supplementary material for: Sharing space at the research table: exploring public and patient involvement in a methodology priority setting partnership
Source: Res Involv Engagem. 2023 May 2;9:29. doi: 10.1186/s40900-023-00438-1 (PMC10152423; doi:10.1186/s40900-023-00438-1)
Supplement: Supplementary file 1 — Additional file 1: Data collection timepoints. Table - List and dates of data collection time points in this case study. [file 40900_2023_438_MOESM1_ESM.pdf]

Appendix 1 – Data collection timepoints

Source of Evidence	Date
**Pre-Meetings Observed**<br>(n=5, 1 hour each, audio recording, transcribed verbatim)	2 Oct-20
	18 Jan-21
	10 Mar-21
	13 May-21
	8 Jun-21
**Steering Group Meetings Observed**<br>(n=4, 1.5 hours each, audio recording, transcribed verbatim)	18 Jan-21
	10 Mar-21
	13 May-21
	9 Jun-21
**Public Partner Focus Groups**<br>(n=2, 1 hour each, audio recording, transcribed verbatim)	12 Mar-21
	26 Jul-21
**Researcher/Methodologist Focus Group**<br>(n=1, 1 hour, audio recording, transcribed verbatim	7 Jul-21
**Public Partner one-to-one interviews**<br>(n=3, up to 1 hour, audio recording, transcribed verbatim)	2 Jul-21
	7 Jul-21
	12 Jul-21
**Researcher one-to-one interviews**<br>(n=3, up to 1 hour, audio recording, transcribed verbatim)	5 Mar-21
	7 Apr-21
	13 Apr-21
**JLA one-to-one interview** (n=1, 30 min, audio recording, transcribed verbatim)	15 Mar-21
**Documentary analysis:** (n=58 total: agendas and minutes of all Steering Group (6) and pre-meetings (7); documents with feedback on the definitions (13), documents with feedback from methodologist and public partner pairs (5); public partners time costing (1), documents with feedback on the video script (3), emails (13) and field notes (10)	Throughout

Appendix 1: List and dates of data collection time points in this case study.
