# Supplementary material for: Sharing space at the research table: exploring public and patient involvement in a methodology priority setting partnership
Source: Res Involv Engagem. 2023 May 2;9:29. doi: 10.1186/s40900-023-00438-1 (PMC10152423; doi:10.1186/s40900-023-00438-1)
Supplement: Supplementary file 2 — Additional file 2: Interview and Focus Group Topic Guide. Semi-Structured one-to-one interview/Focus Group Schedule for Steering Group members of the Priority III PPI qualitative case study. [file 40900_2023_438_MOESM2_ESM.pdf]

Appendix 2 - Interview and Focus Group Topic Guide

Semi-Structured one to one interview/Focus Group Schedule for the Priority III PSP qualitative case study

Introduction:

- Audio-recorded to ensure that key points documented
- Any identifying info (e.g. name or names of others) used during our discussion will be removed
- If you want to end our discussion before I ask all questions or if you want to withdraw from the study you are free to do so.
- The opinions you offer will not impact on your participating in the wider Priority III project

The general aim of the interview is to help us understand your views and experiences of PPI in the Priority III project. There are no right or wrong answers; we are trying to understand what worked and what didn't work, so please answer frankly. Feel free to ask me any questions throughout.

Topic guide

**Summary of proposed interview topics**		
	**Public Partners**	**Researchers/methodologists**
**Role/Expectations**	Did you have clear understanding of what was expected?	Understanding of PPI
	What was your relationship with the research team? Before/during	Expectations of roles of PPI contributors in Priority 3
**Context**	Challenges of being involved – topic, others	Overall experience of PPI in Priority III
**Training and support**	Sufficiently supported to participate? (prompt – training, contact person, payment)<br>What would have helped more?	Training or support given to PPI contributors
**Knowledge**	Benefits of being involved	
**Communication**	Did you feel able to express your views freely?<br>Did you feel your voice was heard?	Experience of language in methodology and PPI
**Impact/influence**	Are you confident that your feedback was taken into consideration?<br>What influence do you think you had to date?<br>Do you feel your input was valued?	Perceived contributions of PPI<br>Challenges of including PPI

- **Are you satisfied or dissatisfied with the involvement?**
- **What are the strengths?**
- **What could be improved?**
- **What else would you like us to know?**

That's all the questions we have; has anything occurred to you about this topic that we haven't asked about?

Thank you!
