# Supplementary material for: Sharing space at the research table: exploring public and patient involvement in a methodology priority setting partnership
Source: Res Involv Engagem. 2023 May 2;9:29. doi: 10.1186/s40900-023-00438-1 (PMC10152423; doi:10.1186/s40900-023-00438-1)
Supplement: Supplementary file 3 — Additional file 3: Codes and subthemes. Table—Example of coding used and subthemes generated in this case study. [file 40900_2023_438_MOESM3_ESM.pdf]

Appendix 5

**Codes**	**Clustering Codes**	**Final Theme**	**Subtheme**
• Researcher: open to feedback and views<br>• Public: space to listen – to learn, build capacity in being able to contribute more meaningfully later on<br><br>• Benefits of pre-meetings for researchers - space to reflect<br><br>• Importance of public partners offering ongoing feedback regarding process<br>• Importance of researchers offering space for feedback. comment from public partners re process (e.g. payments)<br><br>• Space to disagree<br><br>• Space to talk, and space to talk versus time constraints<br><br>• Set a slower pace which prompted reflection	Space to listen<br><br>Space to reflect<br><br>Space for feedback<br><br>Space to disagree<br><br>Space to talk<br><br>Space to reflect	*We need support and space at the table*	SPACE<br>Creating safe space to listen, challenge and learn
• Ways to support the public partners<br>• Attempts to level the playing field<br>• Benefits and challenges of pairs of public and methodologists<br>• Benefits of pre-meetings for public partners<br><br>• Supports need to have purpose / use<br>• Extra supports for individuals (e.g. email, extra reading material)<br>• Iterative and experiential learning<br>• When extra supports no longer needed<br><br>• Balancing the different needs of the public partners (challenging and supports that worked)	Support<br><br>Benefits of support<br><br>Iterative support and learning<br><br>Balancing different needs	*We need support and space at the table*	SUPPORT<br>Define and develop support needed for meaningful involvement

• Purpose of pre-steering group meeting needs to be clear	Iterative personalised support		
• Researchers striving for meaningful and purposeful engagement of public partners<br>• Researchers recognised the need for public partners<br>• Perception from public partners of PPI being valued, receptiveness, acceptance, listening, openness<br>• Challenges in methodology topic – supports needed<br>• Benefits of peer support<br>• Room for improvement – more guidance around pairs; context	Supporting meaningful involvement		
• Benefits of supports – increase in public partners contribution; valued and perceived value<br>• Ease of payment supports, clarity and transparency of payment; needs met, feeling valued	Supporting meaningful involvement, benefits of meaningful involvement		
• Benefits of working as part of a group – not a lone voice, range of experience, sparking ideas, mutual learning	Benefits of group		
