# Supplementary material for: Sharing space at the research table: exploring public and patient involvement in a methodology priority setting partnership
Source: Res Involv Engagem. 2023 May 2;9:29. doi: 10.1186/s40900-023-00438-1 (PMC10152423; doi:10.1186/s40900-023-00438-1)
Supplement: Supplementary file 4 — Additional file 4: Tasks and areas of influence of public partners in the Priority III PSP. Table - Description of tasks and activities that the public partners participated in and influenced throughout the stages of Priority III. [file 40900_2023_438_MOESM4_ESM.pdf]

Appendix 3 Tasks and areas of influence of public partners in Priority III

Description of defined tasks and activities that the public partners were expected to, and did participate in, and areas of influence throughout the stages of Priority III.

Phase	Defined tasks and activities	Areas of influence
**Definitions**		Improved clarity of communication through requesting, reviewing and adapting key definitions used in the project
**Steering Group engagement**	Participating in the Steering Group	Took an early and active role in the Steering Group, including presenting updates
	Reviewing meeting minutes and questions for online surveys, provided feedback on publicity materials	Requested and supported development of video explaining the project to a public audience, including influencing the language, design, and diversity of characters in the video. This provided an accessible, engaging format to disseminate the survey to a wider audience.
**PPI and EDI**	Participated in the "pre-meetings" and email discussions	Requested that the learning from the project in terms of involvement be explored, documented and shared (resulting in this case study)
	Provided a directional compass to ensure public-centredness of project	Requested that the payment framework developed for the project be shared more broadly
	Shared resources including plain language, reporting, involvement in different contexts etc.	Advocated for underserved groups, including ensuring representation of survey questions in the interim survey and top 10
**Recruitment**	Used own networks to recruit public and patient participants	Played a key role in recruitment, with the result of >17% response rate in the two surveys in the patient and public groups
**Surveys**	Reviewed surveys	Influenced the design of the surveys, ensuring clarity and sufficient information to allow meaningful participation
**Formulation of interim list**	Commented on interim list of priorities	Public partners were crucially involved in a critical decision-making process for the project (i.e. what constituted as an evidence uncertainty/unanswered question)
	Reviewed questions when paired with methodologists	Improved clarity of list of questions
**Final prioritisation workshop**	Used own networks to recruit public and patient participants	Used their networks to recruit public and patient participants from North America and Africa for the final consensus workshops
	Two Public Partners from the Steering Group participated in the consensus workshops	At least three of the final "top ten" were strongly influenced by public involvement
**Dissemination**	Contributed to the dissemination strategy and shared the results	Influenced the journal for the protocol changing how public co-authors could have their affiliation independent of an institution
	Reviewed and co-authored the final research questions, protocol and scientific article	
