# Supplementary material for: Sharing space at the research table: exploring public and patient involvement in a methodology priority setting partnership
Source: Res Involv Engagem. 2023 May 2;9:29. doi: 10.1186/s40900-023-00438-1 (PMC10152423; doi:10.1186/s40900-023-00438-1)
Supplement: Supplementary file 5 — Additional file 5: Estimated task breakdown and time costings for the Priority III PSP. Table—Payment schedule and tasks. [file 40900_2023_438_MOESM5_ESM.pdf]

Appendix 4 – Estimated task breakdown and time costings for Priority III

Phase	Task	No. of hours
**Initial Steering Group engagement**	Allow for 6 steering group meetings @ 1.5h per meeting + 1.5h prep	18
	Allow for 6 PPI meetings @ 1h per meeting + 1.5h prep	15
	Review meeting minutes and summary of decisions made including confirmed questions for inclusion in initial online survey	2
**Protocol development**	Comment on protocol draft	2
	Comment on protocol to HRB Open journal	2
**Initial online survey**	Feedback on survey and publicity materials	2
	Take and comment on pilot survey	2
	Read progress reports that will note demographics of respondents and consider any necessary targeted publicity	2
**Formulation of interim list**	Comment on interim list of priorities	2
**Interim survey**	Feedback on survey and publicity materials	2
	Take and comment on pilot survey	2
	Read progress reports to that will note demographics of respondents and consider any necessary targeted publicity	1
	Take and comment on pilot survey	1
	Comment on final shortlist to take to the workshop	2
**Final prioritisation workshop**	Comment on workshop materials for use on the day	1
	Day of workshop (potentially attend workshop)	4
**Dissemination**	Comment on draft of final research questions	1
	Comment on draft James Lind Alliance report	1.5
	Comment on draft scientific report/paper	1.5
	**Total**	64
	Number of days at 7.5h per day	8.5
	**At ST£150 per DAY**	**1280**
	NOTE: all costs stated refer to standard costs - all costs for travel, per diems if required etc will also be paid by Priority III	
